# The emerging role of extracellular vesicles in diabetes and complications: mechanistic insights and translational prospects

**DOI:** 10.3389/fendo.2025.1674028

**Published:** 2025-10-07

**Authors:** Siyuan Liu, Zhicheng Pan, Xuzhuo Chen, Zhenlin Wang, Wei Zhong, Jitao Ling, Yixuan Chen, Panpan Xia, Deju Zhang, Xiao Liu, Peng Yu, Zhen Hu, Jing Zhang

**Affiliations:** ^1^ Department of Endocrinology and Metabolism, The Second Affiliated Hospital, Nanchang University, Nanchang, China; ^2^ The Second Clinical Medical College of Nanchang University, Nanchang, Jiangxi, China; ^3^ The First Clinical Medical College of Nanchang University, Nanchang, Jiangxi, China; ^4^ Department of Anesthesiology, The Second Affiliated Hospital, Nanchang University, Nanchang, China; ^5^ Food and Nutritional Sciences, School of Biological Sciences, The University of Hong Kong, Hong Kong, , Hong Kong SAR, China; ^6^ Department of Cardiology, Sun Yat-sen Memorial Hospital, Sun Yat-sen University, Guangzhou, China; ^7^ Department of Ultrasound, The Second Affiliated Hospital, Nanchang University, Nanchang, China

**Keywords:** exosomes, extracellular vesicles, diabetes mellitus, diabetic complications, biomarkers, cell-cell communication, liquid biopsy, targeted therapy

## Abstract

Extracellular vesicles (EVs), particularly exosomes, have emerged as key players in diabetes pathogenesis, diagnosis, and therapy. They regulate intercellular communication, influence islet function, and contribute to diabetic complications such as retinopathy, nephropathy, and cardiomyopathy. Their potential as liquid biopsy biomarkers and engineered therapeutic carriers—delivering nucleic acids, proteins, or stem cell-derived regenerative signals—offers promising avenues for diabetes management. However, there are some critical challenges in clinical translation. Future research must prioritize (1) scalable GMP-compliant production with rigorous quality control, (2) targeted delivery systems via ligand modification or biomimetic engineering, (3) improved biocompatibility through cargo optimization and stealth coatings, and (4) large-scale clinical trials to validate efficacy and safety. Addressing these hurdles is essential to harness EVs’ full potential and accelerate their transition into mainstream diabetic care.

## 1 Introduction

Diabetes mellitus has been found to be a global metabolic disease and a leading chronic non-communicable disease worldwide, accounting for 9.3% of all-age mortality (WHO, 2023) (1). Diabetes mellitus pathogenesis involves complex interactions between genetic predisposition and modifiable risk factors including obesity (BMI ≥30), physical inactivity (<150 min/week exercise), and poor dietary habits (high glycemic index foods) (2). Chronic hyperglycemia may cause microvascular (including nephropathy, retinopathy, neuropathy) and macrovascular complications (e.g., coronary artery disease, ischemic stroke, peripheral artery disease), which collectively contribute to a 2–4 times higher mortality risk and significant quality-of-life impairment (measured by EQ-5D scores) (3). Diabetes and its complications place a heavy economic and health burden on individuals, families and society. Hence, developing cost-effective prevention paradigms and evidence-based treatment strategies constitutes a public health imperative to achieve UN Sustainable Development Goal 3.4 (reduce NCD mortality) (4).

### 1.1 The importance of intercellular communication in diabetes and its complications

Diabetes mellitus is a systemic metabolic disorder affecting multiple cell types, including islet β-cells, hepatocytes, adipocytes, and vascular endothelial cells (among others) (5). Precise intercellular communication is critical for maintaining metabolic homeostasis and orchestrating responses to diverse physiological challenges and pathological insults (6). Intercellular communication occurs via three primary mechanisms: (1) direct cell-cell contact through gap junctions, (2) paracrine/endocrine signaling molecules, and (3) synaptic neurotransmission (7). In recent years, extracellular vesicles, such as EXOs, have attracted extensive attention as a new mechanism of intercellular signal transmission (8). Exosomes are nanoscale vesicles (30–150 nm) derived from the endosomal system, and are enriched with cargo molecules including proteins, nucleic acids, and lipids that reflect their cell of origin (9). Exosomes facilitate bidirectional molecular transfer across biological barriers, thereby mediating interorgan communication that critically regulates both physiological processes and disease pathogenesis (10). Under diabetic conditions, pancreatic β-cells, hepatocytes, adipocytes, and vascular endothelial cells release distinct populations of exosomes involved in metabolic dysregulation and complication progression through organ-crosstalk mechanisms (11).

### 1.2 The rise of exosomes as a new intercellular communication carrier

As novel intercellular communication carriers, EXOs have garnered considerable attention in diabetes pathophysiology fields, particularly regarding its complications (12). Functionally, EXOs transport cell-specific bioactive cargos (e.g., proteins, nucleic acids) that serve as promising biomarkers for early diagnosis and accurate prognosis prediction (13). Mechanistically, exosome-mediated intercellular communication orchestrates diabetes onset and complication progression, thereby representing a new therapeutic avenue (14). A growing body of evidence confirms the critical role of EXOs in regulating metabolic homeostasis and contributing to the pathogenesis of diabetes (15). Specifically, β-cell-derived EXOs modulate insulin biosynthesis and secretion, whereas hepatocyte- and adipocyte- secreted EXOs fine-tune systemic glucose homeostasis and lipid metabolic fluxes (16). Vascular-cell-derived EXOs are actively involved in diabetes-associated endothelial dysfunction and vascular remodeling via paracrine signaling (17). Immune-cell-derived EXOs mediate low-grade chronic inflammation, a hallmark of diabetes progression (11). Overall, these findings establish a conceptual framework for deciphering the exosome network in diabetes, while paving the way for innovative exosome-centered diagnostic and therapeutic platforms (18). In summary, diabetes and its complications constitute a pressing global health crisis, necessitating the development of novel intervention strategies. As versatile intercellular messengers, EXOs underlie critical aspects of diabetes pathophysiology, thereby offering transformative potential for revolutionizing diabetes management through precision diagnostics and targeted therapies.

### 1.3 Literature search methodology

Databases: PubMed and Web of Science.

Keywords: “extracellular vesicles,” “exosomes,” “diabetes,” “diabetic complications,” “biomarkers,” and “therapeutics.”

Time Range: January 2000–May 2025.

Inclusion Criteria: (1) Original research/reviews on EVs in diabetes; (2) Studies with mechanistic/clinical insights; and (3) English-language publications.

Screening: Titles/abstracts screened for relevance, followed by full-text review.

## 2 Biological characteristics of exosomes and their role in islet cells

### 2.1 Definition and classification of exosomes

EXOs represent nanoscale extracellular vesicles that are actively secreted by virtually all cell types (19). Derived from the endosomal system, EXOs are generated through the inward budding of endosomal membranes, resulting in intraluminal vesicles (ILVs) that accumulate within multivesicular bodies (MVBs) (20). Following MVB-plasma membrane fusion, ILVs are secreted into the extracellular space as EXOs (21). After extracellular release, ILVs acquire the designation of EXOs (22). Exosome cargos incorporate molecular constituents from multiple organelles (e.g., endoplasmic reticulum, Golgi, mitochondria), conferring dynamic functionality that adapts to diabetic conditions and extracellular milieu (23).

Exosomes are classified into two categories: naturally occurring EXOs and engineered EXOs (24). Physiological EXOs are prevalent in various biological fluids and exert pleiotropic regulatory functions (25). For instance, EXOs in breast milk is enriched with developing miRNAs and bioactive lipids that program immune maturation and metabolic adaptation in infants (26). Experimental studies reveal that spp. human milk EXOs exhibit broad-spectrum antimicrobial activities, including direct viral neutralization and bacterial pathogen inhibition (27). Plasma EXOs are the most broadly characterized, derived from various cellular sources including platelets, leukocytes, and malignant cells. Plasma EXOs derived from metabolically active cells (e.g., adipocytes, hepatocytes) mediate systemic inflammation and insulin resistance in diabetes. Their dysregulated cargo profiles (e.g., miR-192, miR-222) serve as clinically validated liquid biopsy biomarkers for diabetic complications (28 –29).

### 2.2 Biogenesis and secretion mechanism of exosomes

Exosome biogenesis is controlled by two major molecular pathways: the ESCRT-dependent and ESCRT-independent mechanisms (21). In addition to the ESCRT mechanism, ESCRT-independent mechanisms have been identified, such as the role of tetraspanins (e.g., CD9) and lipid modifying enzymes (e.g., nSMase2) (30).Exosome secretion requires Rab GTPase-mediated trafficking and SNARE-dependent membrane fusion (31). Hyperglycemia upregulates nSMase2 activity in diabetic models, accelerating pathogenic EV secretion and altering cargo profiles (32, 33). Dysregulated secretion under diabetic oxidative stress contributes to aberrant EV release.

### 2.3 Molecular composition of exosomes

Exosomes possess a complex biochemical profile, encompassing characteristic proteins (e.g.CD9, CD63), nucleic acids (including mRNAs and miRNAs), and specialized lipids (e.g.cholesterol, phosphatidylcholine) (34). Critically, hyperglycemia alters exosomal protein glycosylation patterns (e.g., CD63 sialylation) in diabetes, accelerating endothelial dysfunction (35). These constituents serve as discriminatory markers and mediate biological functions (36). In diabetes, exosomal miR-192-5p and miR-222-3p serve as validated biomarkers for nephropathy progression (37).

Tetraspanins (e.g., CD9/CD63) regulate membrane dynamics and cargo sorting (38). Biogenesis proteins (e.g., ALIX, TSG101) orchestrate cargo loading. ALIX mediates pathological miR-155 sorting in adipocyte-derived exosomes under high-glucose conditions, promoting macrophage inflammation (39).

Nucleic acids (particularly mRNAs and miRNAs) constitute functional exosome cargos that can reprogram recipient cell phenotypes through horizontal gene transfer (40). In diabetic retinopathy, Müller cell-derived exosomal transfers to retinal endothelial cells, triggering VEGF overexpression and angiogenesis (41). Exosomes are enriched in functional mRNAs and miRNAs (42).

**Figure 1:**
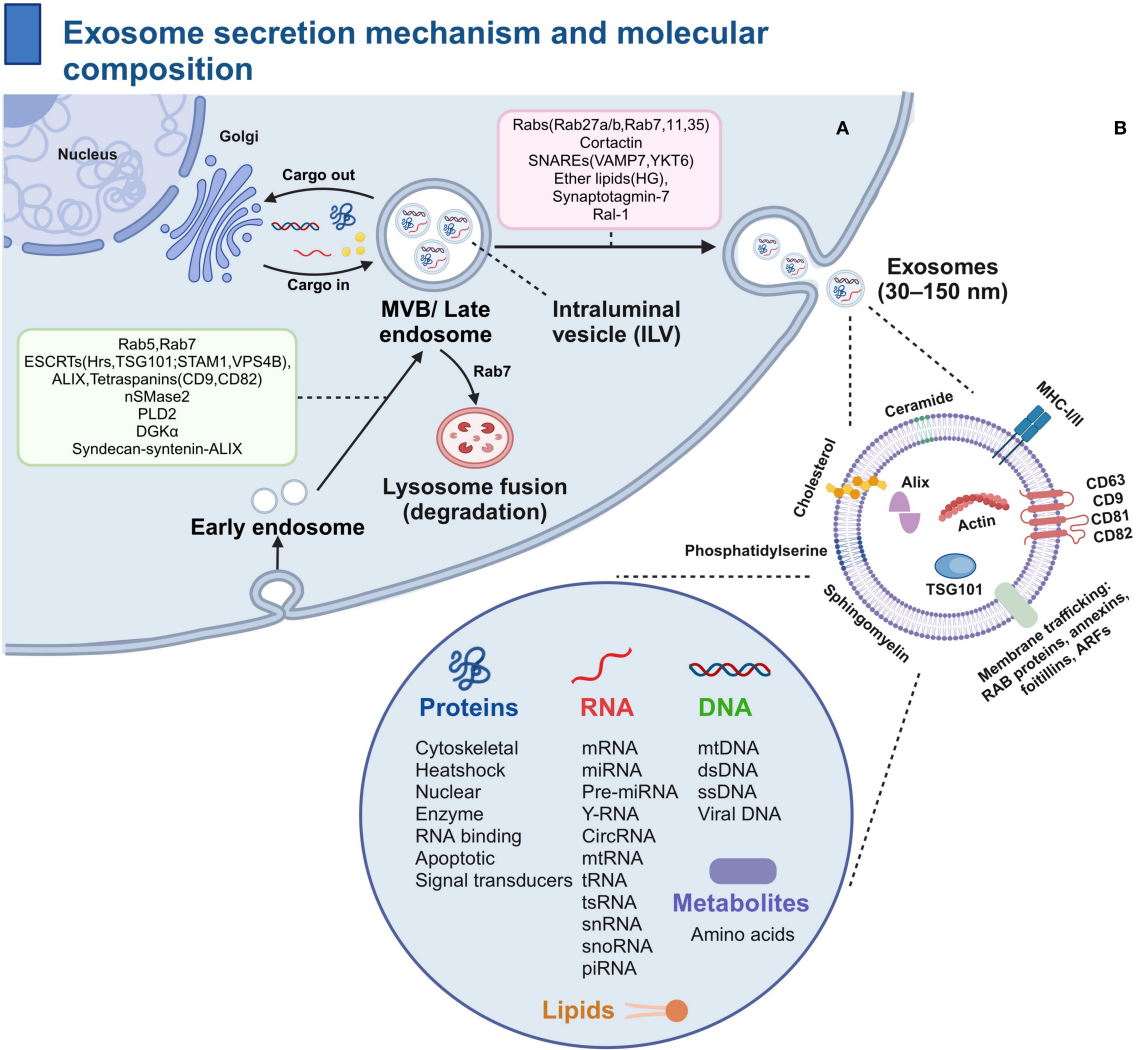
Exosome secretion mechanism and molecular composition: **(A)** Exosome Secretion Mechanism: Cells form early endosomes through endocytosis, which mature into late endosomes or MVBs under the influence of ESCRT and key proteins such as CD9 and nSMase2. ILVs are generated within these structures. Subsequently, EXOs are secreted through cargo loading, transport, and fusion with the plasma membrane, a process regulated by lysosomes. **(B)** Molecular Composition of Exosomes: 1)Proteins: Enriched in tetraspanins (CD9/CD63/CD81) and typically containing ALIX, these proteins are generally regarded as markers for EXOs, 2)Nucleic Acids: mRNA and miRNA are the primary nucleic acids contained within EXOs, mediating the regulation of recipient cell phenotypes, and 3) Lipids: Rich in cholesterol, phosphatidylcholine, and sphingomyelin, these lipids contribute to the integrity of the exosome membrane, resistance to enzymatic degradation, and regulation of the circulating half-life of EXOs. MVB, multivesicular body; ILV, intraluminal vesicle; circRNA, circular RNA.

Lipidome determines exosome membrane integrity and circulating stability. Exosomal lipids are enriched in cholesterol (42.5%), phosphatidylcholine (15.9%), and sphingomyelin (12.5%), forming a unique biochemical signature that differs markedly from parent cells and enhances their drug delivery efficacy (43) (**Figure 1**).

## 3 The role of exosomes in the regulation of islet function and the maintenance of glucose metabolic homeostasis

### 3.1 Exosomes and glucose and lipid metabolism in liver and adipose tissue

EXOs derived from diverse sources contain specific miRNA, including miR-141-3p, miR-29a-3p, miR-210, and miR-1249-3p, as well as proteins and other bioactive molecules. These components have been shown to modulate the expression of genes associated with these metabolic processes, profoundly influencing insulin sensitivity, insulin resistance (IR), and lipid homeostasis.

EXOs from distinct cellular sources regulate hepatic and systemic glucose homeostasis through bioactive molecule delivery. Adipocyte EXOs contain miR-141-3p, which downregulates phosphatase and tensin homolog (PTEN) expression in hepatocytes, thereby promoting glucose uptake (44). EXOs secreted by mesenchymal stem cells (MSCs) activate AMPK in hepatocytes, promoting glycolysis, glycogen synthesis, and inhibiting gluconeogenesis (45). The diabetic microenvironment reprograms the biological functions of EXOs, leading to a disruption of hepatic glucose homeostasis. Diabetic complications including capillary thrombosis, vascular degeneration, and erythrocyte dysfunction establish tissue hypoxia via impairing nutrient delivery (46). Hypoxia induces hypoxia-inducible factor-1α (HIF-1α) expression in hepatic stellate cells (HSCs). Activated HSCs elevate glycolytic flux in quiescent HSCs, Kupffer cells, and liver sinusoidal endothelial cells, collectively enhancing hepatic glucose uptake via EXOs carrying glucose transporter (GLUT) 1 and pyruvate kinase M2 (PKM2) (47). under high-glucose conditions, macrophages increase the loading of miR-210 in EXOs, enhancing the inhibition of NADH dehydrogenase ubiquinone 1 alpha subcomplex (NDUFA) 4 and complex IV (CIV) in adipocytes, thus disrupting their glucose uptake and metabolism (48).

EXOs-mediated intercellular communication coordinates insulin sensitivity by shuttling miR and signaling effectors. During the compensated phase of chronic obesity, hepatocyte EXOs contain miR-3075, which downregulates FA2H expression, stabilizing insulin responsiveness (49). Metabolic stress-induced pathological molecular remodeling directly impairs tissue insulin sensitivity through impaired insulin receptor substrate-1 (IRS1) tyrosine phosphorylation. in obese individuals, adipocyte EXOs show decreased miR-141-3p, leading to upregulation of PTEN in hepatocytes (44). Synchronously, the elevated free fatty acid (FFA) induces pancreatic β-cells to release EXOs containing miR-29. These vesicles are transported to the liver through circulation, where they impair the transduction of insulin receptor signaling pathways (50). Under high-glucose or high-insulin conditions, adipocytes increase the loading of Sonic Hedgehog (Shh) in EXOs, inducing M1 polarization of macrophages via the Ptch/PI3K pathway, and EXOs from M1 macrophages can downregulate IRS-1 and hormone-sensitive lipase (HSL) in adipocytes (51). Importantly, obesity leads to pathologically increased miR-155 levels in adipose tissue macrophage (ATM) EXOs, which inhibit peroxisome proliferator-activated receptor (PPAR)γ expression and AKT activation, thereby reducing insulin responsiveness in hepatocytes and adipocytes (11).

EXOs secreted by various cells transported cell-specific miRs and enzyme to establish a complex molecular network that enhanced or attenuated IR through multiple mechanisms. Adipocyte EXOs contain miR-29a-3p, which inhibits IRS-1 expression and phosphorylation in hepatocytes, enhancing hepatic insulin resistance (52). obesity leads to increased miR-434-3p in hepatocyte EXOs, which promotes M1 macrophage polarization while inhibiting M2 polarization, causing sustained hepatic inflammation and disruption of IRS-1 phosphorylation (49). Conversely, EXOs from pancreatic β-cells are enriched with neutral ceramidase (NCDase), which inhibits FFA-induced oxidative stress and alleviates IR (53). MSCs-derived EXOs significantly enhance IRS-1 expression and ameliorate adipocyte IR by activating the PI3K/Sirtuin1 (SIRT1) axis (54). EXOs from NK cells contain elevated miR-1249-3p, which silences the SKI family transcription corepressor 1 (SKOR1) gene, thereby reducing TLR4/nuclear factor (NF)-κB pathway-mediated IR (55). Aging results in increased miR-29b-3p in BMSCs-EXOs, which inhibit SIRT1 expression, promoting IR in hepatocytes and adipocytes (56).

**Figure 2:**
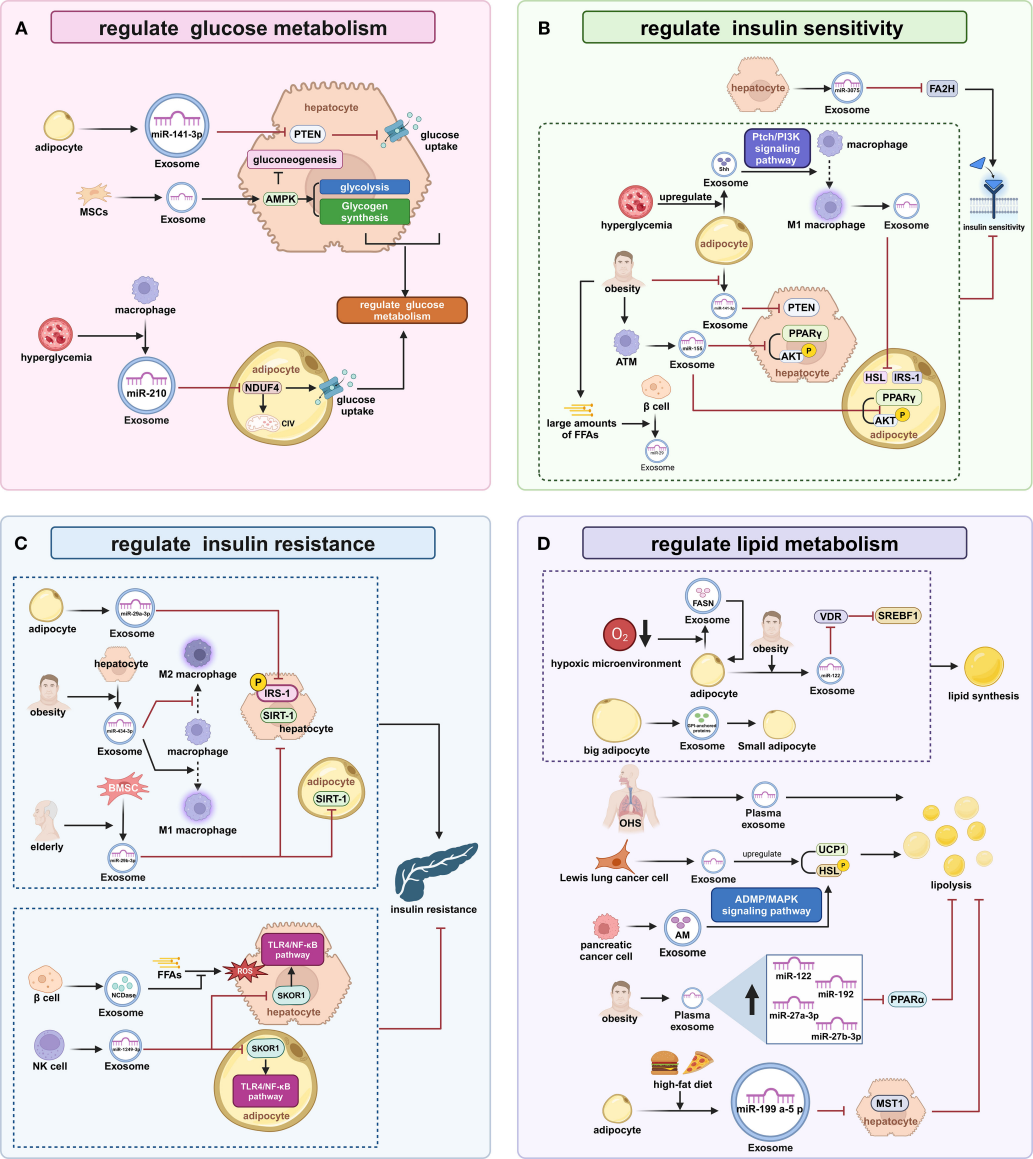
The Regulation of Glucose and Lipid Metabolism in the Liver and Adipose Tissue by EXOs **(A)** Regulation of Glucose Metabolism: Adipocyte exosomes reduce hepatocyte PTEN via miR-141-3p to promote glucose uptake; MSCs exosomes activate hepatocyte AMPK enhancing glycolysis and glycogen synthesis while suppressing gluconeogenesis; Macrophage exosomes under high glucose inhibit adipocyte NDUFA4/CIV with miR-210 disrupting glucose metabolism. **(B)** Regulation of Tissue Insulin Sensitivity: Hepatocyte exosomes during obesity downregulate FA2H via miR-3075 stabilizing insulin response; Obese adipocyte exosomes elevate hepatocyte PTEN due to reduced miR-141-3p impairing insulin sensitivity;β-cell exosomes release miR-29 suppressing insulin responsiveness under high FFAs; Adipocyte exosomes load Shh driving M1 polarization through Ptch/PI3K signaling;M1 macrophage exosomes downregulate adipocyte IRS-1 and HSL; Obese ATM exosomes inhibit PPARγ/AKT with miR-155 reducing insulin sensitivity. **(C)** Regulation of Tissue Insulin Sensitivity: Adipocyte exosomes suppress hepatic IRS-1 expression via miR-29a-3p; Obese hepatocyte exosomes promote M1 polarization with miR-434-3p disrupting IRS-1 phosphorylation; Aging BMSC exosomes inhibit SIRT1 via miR-29b-3p exacerbating insulin resistance; β-cell exosomes mitigate FFA-induced oxidative stress using NCDase alleviating IR; NK-cell exosomes silence SKOR1 with miR-1249-3p inhibiting TLR4/NF-κB-mediated IR. **(D)** Regulation of Lipid Metabolism: Adipocyte exosomes after high-fat feeding suppress hepatocyte MST1 via miR-199a-5p inhibiting β-oxidation; Hypoxic adipocyte exosomes promote lipogenesis through FASN enrichment; Hypertrophic adipocyte exosomes stimulate fatty acid esterification via GPI transfer; OHS plasma exosomes promote lipolysis; Obese plasma exosomes suppress β-oxidation by miR-122 inhibiting PPARα;Obese adipocyte exosomes enhance lipogenesis via miR-122 reducing VDR and activating SREBF1;Lewis lung carcinoma cells EXOs increase lipolysis by upregulating UCP1/HSL; Pancreatic cancer exosomes activate HSL via adrenomedullin promoting lipolysis. PTEN, phosphatase and tensin homolog; MSCs, Mesenchymal stem cells; AMPK, AMP-activated protein kinase; NDUFA, NADH dehydrogenase ubiquinone 1 alpha subcomplex; CIV, complex IV; PPAR, peroxisome proliferator-activated receptor; AKT, protein kinase B; HSL, hormone-sensitive lipase; IRS1, insulin receptor substrate-1; ATMs, adipose tissue macrophages; FFA, free fatty acid; SIRT1, Sirtuin1; ROS, reactive oxygen species; SKOR1, SKI family transcriptional corepressor 1; OHS, Obstructive hypoventilation syndrome; UCP1, uncoupling protein 1; HSL, hormone-sensitive lipase; MST1, mammalian sterile 20-like kinase 1.

EXOs orchestrate systemic lipid homeostasis via interorgan communication networks that transports bioactive substances, including miRs, rate-limiting enzymes, and signaling mediators that dynamically regulate lipogenesis-lipolysis balance. Chronic high-fat feeding triggers packaging of miR-199a-5p into adipose-derived EXOs, which bind to the mammalian sterile 20-like kinase 1 (MST1) in hepatocytes, resulting in marked suppression of fatty acid β-oxidation (57). hypoxia increases the loading of FASN in adipocyte EXOs, promoting *de novo* lipogenesis (58). hypertrophic adipocytes secrete EXOs containing glycosylphosphatidylinositol (GPI), which promote fatty acid esterification in small adipocytes (59). circulating EXOs from individuals with Obstructive hypoventilation syndrome (OHS) promote lipolysis (60). in obese individuals, plasma EXOs show increased levels of miR-122, which inhibits PPARα, thereby suppressing fatty acid β-oxidation (61). adipocyte EXOs from obese individuals contain higher levels of miR-122, which inhibits VDR expression, thereby reducing its suppression of the SREBF1 promoter and enhancing lipogenesis (62, 63). Certain tumor cells remodel lipid metabolism through EXOs secretion (64). EXOs from Lewis lung carcinoma cells upregulate upregulating uncoupling protein 1 (UCP1) and HSL in adipocytes, thereby promoting lipolysis (65). EXOs from pancreatic cancer cells are enriched with adrenomedullin (AM), which activates HSL via the adrenomedullin receptor (ADMR)/MAPK pathway, thereby promoting lipolysis (66) (**Figure 2**).

EXOs regulate liver-adipose glucose-lipid homeostasis by delivering miRNAs, enzymes, and signaling molecules. These vesicles exhibit functional heterogeneity. Some enhance insulin sensitivity and nutrient utilization, while others exacerbate metabolic dysfunction. Systemic metabolic balance relies on equilibrium between these opposing EXO actions. Obesity and hyperglycemia disrupt this balance, inducing pathogenic EXO overproduction that amplifies tissue metabolic disorders. Research on EXO-mediated liver-adipose crosstalk is essential for elucidating metabolic disease mechanisms and developing therapies.

### 3.2 The role of exosomes-mediated intercellular communication in the regulation of islet cell function

EXOs are a key endocrine regulator of β-cell homeostasis, orchestrating cellular proliferation, apoptosis, and insulin secretion through their cargo of cell-specific miR, proteases, and metabolic modulators.

Multiple types of EXOs affect β-cell apoptosis. Gestational diabetes mellitus (GDM) placental EXO contains elevated miR-320b, which induces β-cell apoptosis (67). Immune cells are also involved in the regulation of β-cell function. in type 1 diabetes mellitus (T1DM), islet cell EXOs contain antigens such as glutamic acid decarboxylase 65 and insulin, which mediate immune responses against pancreatic β-cells by T and B cells. T-cell EXOs are enriched with miR-142-3p and other miRNAs closely related to β-cell apoptosis (68). In a related study, EXOs secreted by T cells were found to contain miR-142-3p, miR-142-5p, and miR-155. these miRNAs can activate specific programmed cell death pathways in β-cells, which is associated with the pathogenesis of T1DM (69). The EXOs released by endothelial progenitor cells may contain miR-126 and miR-296, which play a role in promoting insulin secretion by the pancreas, inhibiting β-cell apoptosis, and promoting intra-islet angiogenesis. Therefore, these EXOs have a positive significance for the maintenance of the structure and function of the islets (70).

EXOs regulate β-cell proliferation through multiple pathways. Omentin-1, a protein found in blood exosome-like vesicles (ELVs), has been shown to promote β-cell division (71). It has been reported that bone marrow transplantation increases the levels of miR-106b-5p and miR-222-3p in recipient bone marrow cell EXOs, which downregulate cyclin-dependent kinase inhibitor 1A and 1B in β-cells (72). High-fat stimulation induces IR in skeletal muscle cells, increasing miR-16 in their EXOs, which inhibits Ptch1 expression in β-cells (73). MSCs EXOs can upregulate genes such as pancreatic and duodenal homeobox (Pdx) 1 in the pancreas (74). EXOs from umbilical cord blood MSCs promote β-cell proliferation through the Exostosin-like (Extl)3- regeneration protein (Reg)-cyclinD1 signaling pathway (75).

**Figure 3:**
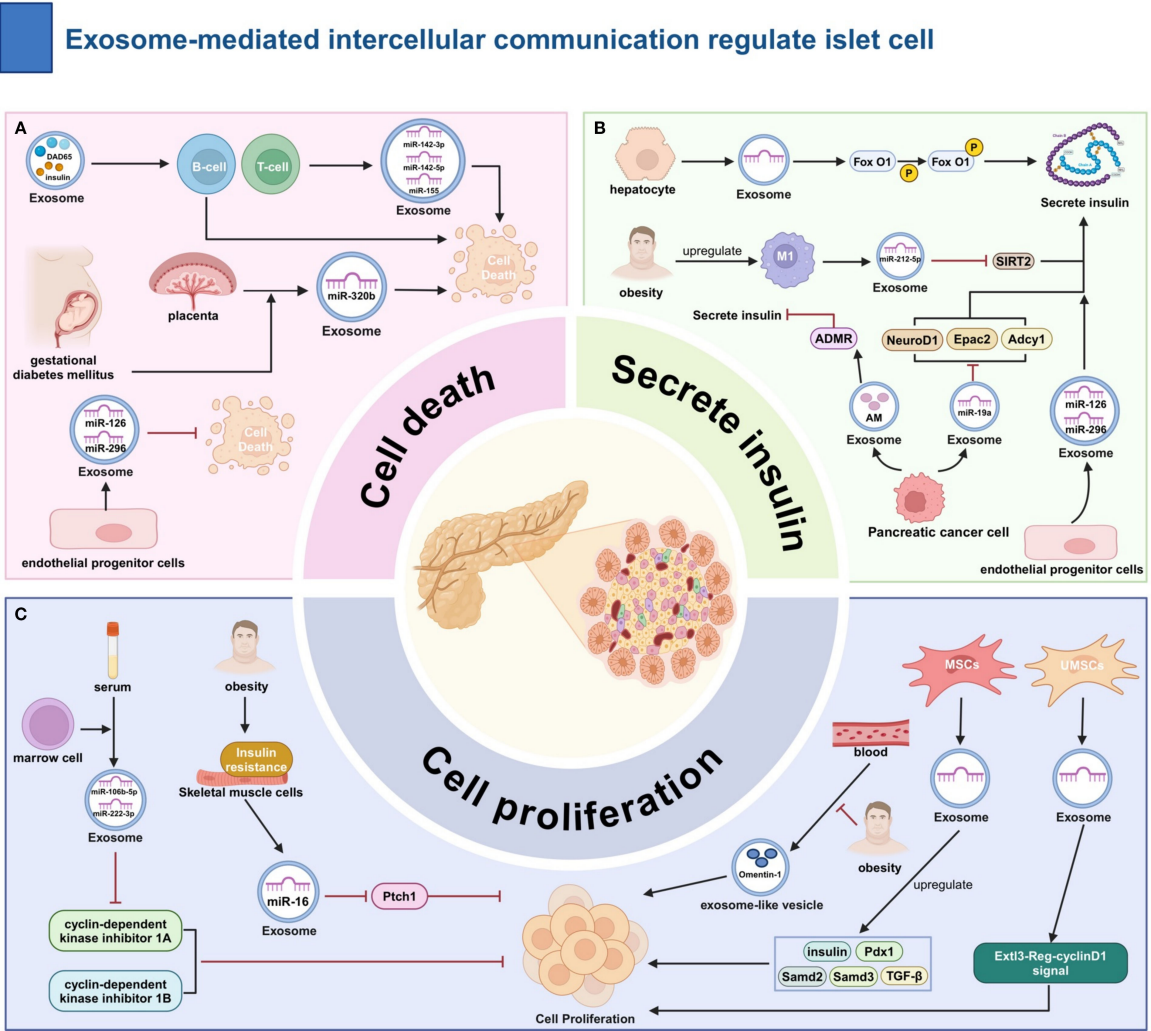
The Role of Exosome-Mediated Intercellular Communication in the Regulation of Islet Cell Function. **(A)** Regulation of Apoptosis: Pro-apoptotic: GDM placenta EXOs (miR-320b); T1DM islet EXOs (autoantigens); T-cell EXOs (miR-142-3p). Anti-apoptotic: Endothelial progenitor EXOs. **(B)** Regulation of Insulin Secretion: Inhibition:M1 macrophage EXOs (miR-212-5p ↓SIRT2); Pancreatic cancer EXOs (miR-19a ↓Neurod1/Adcy1/Epac2; AM→ADMR).Promotion: Hepatocyte EXOs (p-FoxO1); Endothelial progenitor EXOs (miR-126/296). **(C)** Promotion of β-cell Proliferation: Bone marrow EXOs (↑miR-106b-5p/222-3p ↓CDKN1A/B); Muscle cell EXOs (↑miR-16 ↓Ptch1); MSCs EXOs (↑e.g., Pdx1); Blood ELVs (Omentin-1); UC-MSCs EXOs (Extl3-Reg-cyclinD1). ADMR, adrenomedullin receptor; Epac, exchange protein activated by Camp;ET-1, endothelin-1; Adcy, adenylate cyclase; Ptch, Patched; MSCs, Mesenchymal stem cells; Pdx, pancreatic and duodenal homeobox; Samd, Sterile Alpha Motif Domain Containing; TGF-β, Transforming growth factor beta.

The insulin secretion function of β-cells is widely regulated by EXOs. High-fat stimulation increases the number of M1 macrophages in the islets, which secrete EXOs rich in miR-212-5p, inhibiting SIRT2 expression in β-cells (76). The impact of pancreatic cancer on islet function may involve the participation of EXOs. EXOs from pancreatic cancer cells contain miR-19a, which inhibits the expression of neuronal differentiation (Neurod)1, adenylate cyclase (Adcy)1, and exchange protein activated by cAMP (Epac)2 in β-cells (77). In addition, AM in pancreatic cancer cell EXOs acts on the ADMR of β-cells to inhibit insulin secretion (78). Hepatocytes in a state of low insulin sensitivity can promote the phosphorylation of the transcription factor FoxO1 within β-cells by secreting EXOs, thereby enhancing the insulin secretion function of β-cells (79). EXOs secreted by bone marrow-derived MSCs are loaded with miR-146a, which promotes the differentiation of hypo-differentiated β-cells in T2DM. In addition, they enhance β-cell function by regulating the Numb homolog (Numb)/β-catenin pathway (80) (**Figure 3**).

In summary, EXOs have a dual role in the regulation of islet function as an important medium of communication between islet cells and other tissue cells, depending on the source of the EXOs and the specific bioactive substances they carry. In-depth research on the molecular mechanisms of exosome interactions with islet cells will help to elucidate the pathogenesis of diabetes and provide new insights for the prevention and treatment of diabetes.

## 4 Role of exosomes in the development of diabetes and its complications

### 4.1 The exosomes and diabetic nephropathy

Exosomes play a key role in the inflammatory pathogenesis of diabetic nephropathy (DN) (81). Exosomes orchestrate renal inflammation via shuttling pro-inflammatory mediators, including tumor necrosis factor-α (TNF-α), specific interleukins (e.g., IL-1β, IL-6), and their regulatory RNAs (82). Urinary EXOs from diabetic patients are enriched with inflammatory cargos that potentiate immune activation in both tubular epithelial cells and glomerular podocytes, amplifying intrarenal inflammation. Notably, exosomal TGF-β1 has been mechanistically linked to both renal inflammatory signaling and fibrotic cascades (83). Hyperglycemia induced by diabetes stimulates renal cells to release EXOs that contain fibrotic factors, such as transforming growth factor β (TGF-β) and matrix metalloproteinases (MMPs). The factors carried by these EXOs can activate renal interstitial cells and promote collagen deposition, ultimately leading to fibrosis of both renal tubules and glomeruli. There is evidence that TGF-β1 released by EXOs is positively correlated with the degree of fibrosis in animal models of diabetes, and miRNAs such as miR-21 contained in EXOs are also associated with fibrosis (84). Exosomes reflect the pathological status and progression of diabetic nephropathy through the specific molecules they transport. For example, exosomal miRNAs (e.g., miR-21, miR-192) and protein markers (e.g., TGF-β1, TNF-α) in urine are regarded as potential biomarkers for diabetic nephropathy. Studies have shown that the levels of these exosomal components correlate with the severity and progression of diabetic nephropathy, providing critical insights for early diagnosis and disease monitoring (14).

### 4.2 The exosomes with diabetic retinopathy

Chronic low-grade inflammation is a hallmark of diabetic retinopathy (DR) pathogenesis and disease progression. Exosomes mediate intercellular transfer of pro-inflammatory mediators, including tumor necrosis factor-α (TNF-α), specific interleukins (e.g., IL-1β, IL-6), and regulatory miRNAs (e.g., miR-21-5p), thereby amplifying inflammatory cascades in retinal endothelial cells and retinal pigment epithelium (RPE). Clinical studies demonstrate that diabetic retinal EXOs are enriched with TNF-α and IL-1β, which activate NF-κB signaling, leading to disruption of blood-retinal barrier (BRB) integrity and aggravation of retinal pathology (85). Exosomes heavily regulate pathologic retinal neovascularization and vascular dysfunction in the DR. In diabetic conditions, exosomal VEGF levels are significantly upregulated, leading to the formation of leaky, immature neovessels that frequently bleed due to lack of pericytes and disruption of endothelial connections. Experimental evidence reveals that exosome-mediated vascular malformations via VEGF signaling exhibit a strong correlation with DR severity in diabetic rodent models (86). Exosomes represent promising liquid biopsy tools for DR diagnosis and prognostic stratification. Ocular exosomes contain disease-specific biomarkers (e.g., VEGF-A, TGF-β1, miR-21-5p) that allow for noninvasive monitoring and molecular classification of DR. Longitudinal studies confirm that quantitative changes in these exosomal biomarkers reflect DR staging and predict disease progression, facilitating timely therapeutic interventions (27). According to the latest research findings, these vesicles exhibit different molecular characteristics when comparing the extracellular vesicles in the vitreous of diabetic individuals and non-diabetic individuals. Among them, Cldn5 (an endothelial tight junction protein) increased significantly. The upregulation of Cldn5 in the vitreous exosomes of diabetic patients indicates its potential endothelial origin. Cldn5 is associated with endothelial dysfunction and increased vascular permeability (87).

### 4.3 Exosomes and diabetic neuropathy

The onset of DN is strongly associated with various factors, including hyperglycemia, oxidative stress, inflammatory responses, and alterations in nerve growth factor (NGF) levels. These factors contribute to neuronal damage and dysfunction, ultimately resulting in alterations in sensory, motor, and autonomic functions. Exosomes have been identified as carriers of inflammatory molecules, such as TNF-α and IL-6, facilitating intercellular communication of these factors. Hyperglycemia, a hallmark of diabetes, triggers the release of inflammatory molecules within EXOs, thereby amplifying the neuroinflammatory response. This response accelerates neuronal damage and exacerbates the progression of diabetic neuropathy (88). In the diabetic environment, where levels of oxidative stress are elevated, EXOs propagate oxidative stress by carrying oxidative markers, including hydrogen peroxide and oxidized lipids. These EXOs induce intracellular redox imbalance, impairing neuronal structure and function and leading to worsening neuropathy (89). Additionally, EXOs in diabetic patients may also transport neuroprotective factors such as brain-derived neurotrophic factor (BDNF) and glial cell-derived nerve growth factor (GDNF). Disruption of the transport of these factors may hinder nerve cell repair and regeneration, further exacerbating the severity of neuropathy (90).

## 5 The role of exosomes in diabetic cardiomyopathy

### 5.1 Exosomes and diabetic myocardial apoptosis and necrosis

Exosomes play a critical role in the pathogenesis of cardiomyocyte apoptosis and necrosis in diabetic cardiomyopathy (91). Diabetes induces elevated oxidative stress and inflammation in cardiomyocytes, which are communicated between cells through exosome-mediated transfer (92). Under diabetic conditions, EXOs secreted by cardiomyocytes are enriched with pro-apoptotic factors, such as Caspase-3 and Smac/DIABLO, which contribute to the activation of apoptotic pathways (93). Exosomes also influence cardiomyocyte survival by carrying and delivering miRNAs such as miR-21. miR-21 can indirectly enhance the apoptotic process by targeting and inhibiting apoptosis inhibitors such as PTEN (94). Moreover, inflammatory factors in EXOs, such as TNF-α and IL-6, can activate the NF-κB signaling pathway, enhance the expression of apoptosis-related proteins, and exacerbate cardiomyocyte apoptosis and necrosis (95). Therefore, EXOs promote apoptosis and necrosis of cardiomyocytes in diabetic cardiomyopathy through multiple mechanisms (92).

### 5.2 Exosomes and diabetic myocardial remodeling and fibrosis

A hallmark feature of diabetic cardiomyopathy is myocardial remodeling and fibrosis. Exosomes play a critical regulatory role in these pathological processes. Hyperglycemia and oxidative stress, both hallmarks of diabetes, alter the composition of EXOs, leading to a combination of factors that promote fibrosis. Previous studies have demonstrated that EXOs released by diabetic cardiomyocytes are enriched with TGF-β1 and collagen, and promote myocardial fibrosis by activating the proliferation and collagen synthesis of cardiac fibroblasts (30). Concurrently, miR-21 in EXOs has been shown to exacerbate myocardial fibrosis by upregulating the expression of fibrosis-related genes, such as Col1A1 and Col3A1. Moreover, exosomes modulate myocardial remodeling by regulating the activity of extracellular matrix (ECM) remodeling enzymes, such as matrix metalloproteinases (MMPs), thereby contributing to a further decline in cardiac function. In conclusion, EXOs promote myocardial remodeling and fibrosis in diabetic cardiomyopathy through multiple mechanisms (96).

### 5.3 Exosomes and diabetic myocardial regeneration and repair

There is complex role of EXOs in myocardial regeneration and repair in diabetic cardiomyopathy. Although EXOs in the diabetic state contain factors such as VEGF and basic fibroblast growth factor (bFGF) that promote angiogenesis and cellular repair, they are usually impaired in the diabetic state (97). The changes in diabetic EXOs can affect the function of cardiac stem cells. Diabetic EXOs, for instance, may contain high levels of pro-apoptotic miRNAs (e.g., miR-34a), which inhibit the proliferation and differentiation of stem cells, thereby reducing cardiac regeneration. Moreover, EXOs in the diabetic state may affect the regeneration of cardiomyocytes, thereby hindering myocardial repair by reducing stem cell migration and the release of regulatory factors. Thus, although EXOs play a crucial role in myocardial repair, the effects of diabetes significantly limit their regenerative capacity (98).

### 5.4 Exosomes and diabetic peripheral artery disease

**Figure 4:**
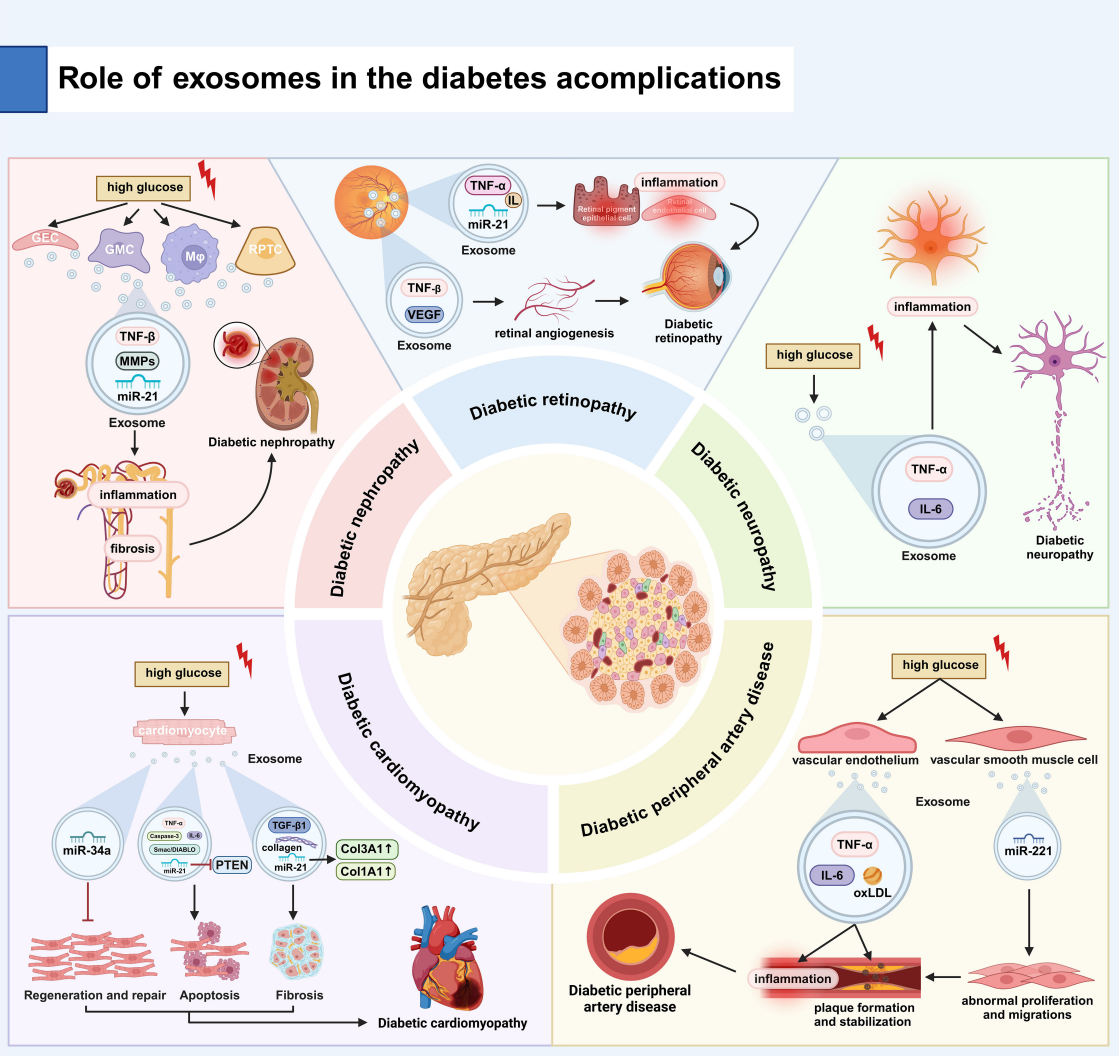
Role of EXOs in the development of diabetes and its complications. **(A)** Diabetic Nephropathy: High glucose stimulates renal cells (GECs, GMCs, RPTCs) to release EXOs carrying TNF-β, MMPs, and miR-21, promoting renal inflammation and fibrosis. **(B)** Diabetic Retinopathy: EXOs deliver TNF-β, IL, and miR-21 to retinal cells, causing inflammation and pathological angiogenesis (via VEGF), leading to leaky vessels. **(C)** Diabetic Neuropathy: High glucose induces EXO-mediated transport of TNF-α/IL-6, driving neuroinflammation and neuronal damage. **(D)** Diabetic Cardiomyopathy: Apoptosis: EXOs contain elevated Caspase-3, miR-21 (suppresses PTEN), and miR-34a, promoting cardiomyocyte death. Fibrosis: EXOs carry TGF-β1, collagen, and miR-21, activating fibroblasts and collagen synthesis. (E) PAD in Diabetes: Endothelial EXOs contain TNF-α, IL-6, and oxLDL, driving vascular inflammation. VSMC EXOs deliver miR-221, inducing pathological VSMC proliferation/migration and atherosclerosis. TNF-α, tumor necrosis factor-α; IL, interleukin; PTEN, phosphatase and tensin homolog; oxLDL, oxidized low-density lipoprotein..

The role of EXOs in diabetes and its complications, particularly peripheral arterial disease (PAD), has garnered increasing attention. Exosomes play a crucial role in the pathogenesis and progression of diabetes and PAD by influencing islet β-cell function, insulin resistance (IR), vascular endothelial function, inflammatory responses, and vascular smooth muscle cell activity. Exosomes are essential for the function and survival of pancreatic β-cells. Exosomes derived from pancreatic β-cells in diabetic patients may carry distinct proteins and miRNAs that could disrupt insulin synthesis and secretion. Studies have demonstrated an increase in exosomal miR-146a levels in islet β-cells of diabetic patients, which can impair β-cell function by inhibiting the insulin signaling pathway (99). Exosomes are implicated in the development of IR. Exosomes in diabetic patients may carry miRNAs associated with the insulin signaling pathway, such as miR-21, which can disrupt the insulin signaling cascade by regulating target gene expression, thus promoting the development of IR (100). The progression of peripheral arterial disease (PAD) is closely associated with endothelial dysfunction (101). Exosomes secreted by vascular endothelial cells in diabetic patients can transport pro-inflammatory factors and oxidative stress markers, which exacerbate endothelial damage. Studies indicate that vascular endothelial EXOs in diabetic patients contain elevated levels of oxidized low-density lipoprotein (oxLDL), which promote endothelial apoptosis and inflammatory responses, contributing to increased arterial stiffness (102). Exosomes in diabetic patients are enriched with inflammatory mediators such as TNF-α and IL-6. these factors contribute to the development of PAD by activating inflammatory pathways in the peripheral vascular system, promoting inflammation, and facilitating plaque formation in the arterial wall (103). In PAD, alterations in vascular smooth muscle cell function play a significant role. Exosomes secreted by vascular smooth muscle cells in diabetic patients may carry altered miRNAs, such as miR-221, which regulate smooth muscle cell proliferation and migration. Abnormal proliferation and migration of smooth muscle cells contribute to the formation and stabilization of atherosclerotic plaques, thereby exacerbating PAD progression (104) (**Figure 4**, **Table 1**).

**Table 1:** EXOs functional mechanism of action.

Disease	Correlation Factor	Mechanism of Action	Study Type
Diabetic Nephropathy (105)	TNF-α, ILs, Related mRNA and miRNA(e.g.miR-21, miR-192)	Immune activation in renal tubular epithelial cells and glomerular podocytes potentiates local inflammatory cascades, as exemplified by exosomal TGF-β1-mediated concurrent inflammation and fibrotic responses.	Animal study
Diabetic Retinopathy (106)	TNF-α, ILs, Inflammation related miRNAs (e.g., miR-21)	Retinal-derived EXOs exacerbate pathological changes by activating the NF-κB signaling pathway, which enhances retinal inflammation and disrupts vascular integrity, ultimately aggravating retinal lesions (107). Retinal-derived EXOs orchestrate pathological angiogenesis by modulating TGF-β and VEGF signaling pathways, leading to aberrant vascular formation and compromised blood-retinal barrier integrity.	Clinical study
Diabetic Neuropathy (108)	TNF-α, IL-6	EXOs propagate oxidative stress by transporting reactive oxygen species and lipid peroxidation products (e.g., 4-HNE, MDA), which disrupt cellular redox homeostasis, impair neuronal integrity, and exacerbate neuropathological progression.	Clinical study
Diabetic Cardiomyopathies (109, 110)	Proapoptotic factors (Caspase-3 and Smac/DIABLO), miRNAs (e.g., miR-21), inflammatory factors (e.g. TNF-α and IL-6), factors that promote myocardial repair (VEGF, bFGF), pro apoptotic miRNAs (e.g., miR-34a)	Cardiac-derived EXOs activate the NF-κB signaling pathway, thereby inducing cardiomyocyte apoptosis through upregulation of pro-apoptotic factors (e.g., Bax, caspase-3) and promoting necrotic cell death via mitochondrial dysfunction. Pathological EXOs impair cardiac regeneration by suppressing stem cell chemotaxis and inhibiting paracrine secretion of reparative factors (VEGF, IGF-1).	Animal study
Diabetic Peripheral Arterial Disease (104)	Different proteins and miRNAs (miR-146a, miR-21), TNF - α, IL-6	Pathological EXOs contribute to metabolic disorders through multifaceted mechanisms: 1. Impairing pancreatic β-cell insulin secretion. 2. Exacerbating insulin resistance in target tissues. 3. Compromising endothelial nitric oxide bioavailability. 4. Amplifying pro-inflammatory cytokine release. 5. Promoting vascular smooth muscle cell phenotypic switching.	Clinical study

EXOs, exosomes; TNF-α, tumor necrosis factor-α; IL, interleukin; TGF-β, Transforming growth factor beta; bFGF, basic fibroblast growth factor; NF, nuclear factor.

## 6 Exosomes as liquid biopsy biomarkers and therapeutic applications in diabetes and its complications

### 6.1 The application of exosomes in the diagnosis of diabetes

Exosomal proteomic and transcriptomic analyses demonstrate considerable potential for application in diabetes diagnosis and pathological mechanism investigation. The bioactive components encapsulated within EXOs (e.g., nucleic acids, proteins, miRNAs, and lncRNAs) exhibit precise correlations with pathological progression, positioning them as promising candidates for minimally invasive diabetes biomarkers.

Emerging evidence suggests that miR-1 and miR-133 expression levels are significantly elevated in prediabetic patients with type 2 diabetes. Notably, these circulating miRNAs exhibit strong diagnostic validity as emerging biomarkers for early-stage T2DM progression (111). Comparative analyses in GDM studies have demonstrated that EXOs derived from umbilical cord blood from affected patients showed increased vesicle diameter and elevated particle concentration compared to healthy pregnant controls (112, 113). Plasma-derived EXOs from GDM-affected pregnancies demonstrate differential expression of specific miRNAs compared to non-GDM counterparts. miR-423-5p shows upregulated expression, while miR-122-5p, miR-148a-3p, miR-192-5p, and miR-99a-5p exhibit concurrent downregulation. These differentially expressed miRNAs demonstrate a predictive capacity for early GDM detection through systematic bioinformatic evaluation (107). A longitudinal study systematically examined placental EXOs in maternal urine to compare miRNA expression profiles (miR-16-5p, miR-222-3p, miR-516-5p, miR-517-3p, and miR-518-5p) between patients with GDM and pregnancies with normal blood glucose. First-trimester analysis revealed no significant intergroup differences. In mid-pregnancy, the expression of all five miRNAs was significantly increased in the GDM cohort, whereas it was significantly down-regulated in the late-pregnancy samples compared to the control group. The diagnostic evaluation identified miR-16-5p, miR-517-3p, and miR-518-5p as high-accuracy biomarkers, with the diagnostic value of the remaining two miRNAs unassessed. This temporal expression pattern suggests the potential utility of placental EXOs biomarkers for preliminary GDM screening (114). Recent studies suggest that visceral adipose tissue volume may significantly contribute to GDM development by modulating miRNA-148 expression in adipose tissue-derived EXOs, highlighting its potential as a screening target for GDM (115). A recent study demonstrated that EXOs derived from the umbilical cord blood of GDM patients exhibit significant alterations in the expression levels of multiple mRNAs and lncRNAs. These lncRNAs may modulate gene expression by acting as miRNA sponges (113). The H19-lncRNA content in serum EXOs is significantly elevated in T2DM patients, whereas MALAT1-lncRNA levels are reduced. These dysregulated nucleic acids may contribute to T2DM pathogenesis and demonstrate potential predictive value for the disease (116). The expression levels of multiple circular RNAs (circRNAs), particularly circ_0033104, circ_0004561, and circ_0058247, were markedly dysregulated in EXOs derived from umbilical cord blood of GDM patients. These circRNAs may mediate miRNA activity via the competitive endogenous RNA (ceRNA) mechanism (112).

### 6.2 Engineered exosome-based therapeutic strategies for diabetes

#### 6.2.1 Surface modification strategies

Chronic excessive inflammatory responses can lead to tissue damage and delay the wound healing process. To mitigate this pathological process, recent studies have developed genetically modified cells that, upon interferon-γ stimulation, produce EXOs enriched with programmed death ligand 1 (117). The programmed death ligand 1 displayed on these extracellular vesicles exhibits a strong binding affinity for programmed death receptor 1 expressed by T lymphocytes. This interaction potently suppresses both T cell activation and expansion, resulting in significant immunosuppressive activity (117). Additionally, these modified extracellular vesicles enhance the migratory capacity of both keratinocytes and dermal fibroblasts, thereby significantly improving tissue regeneration and wound closure rates (117). Thus, this extracellular vesicle-based therapy represents a promising strategy for simultaneous immunomodulation and tissue regeneration, offering new solutions for inflammation control and wound healing.

#### 6.2.2 Loading therapeutic nucleic acid molecules

By employing genetic modification approaches, investigators have engineered parental cells to express specialized proteins capable of molecular recognition and cargo transport. These proteins utilize intrinsic localization signals to mediate the efficient packaging of target molecules into extracellular vesicles, substantially enhancing their intravesicular concentrations (118). This breakthrough demonstrates the considerable potential for engineering extracellular vesicles as targeted drug delivery systems. These findings are particularly important given the established association between systemic chronic inflammation and the pathogenesis of diabetes and its subsequent complications (119). To overcome this challenge, it was shown that extracellular vesicles of bone marrow-derived macrophages were enriched with anti-inflammatory miRNAs (miR-99a, miR-146b, and miR-378a) capable of modulating TNF-α and NF-κB signaling pathways. Interleukin-4 stimulation markedly upregulates these regulatory miRNA, revealing novel therapeutic opportunities for inflammation control. These findings are particularly relevant to diabetic foot ulcers, a common complication of diabetes mellitus that is closely associated with progressive lower extremity atherosclerosis (118). Enhancement of low-density lipoprotein receptor (LDLR) expression is a promising therapeutic strategy to mitigate the progression of atherosclerosis. Elevated levels of LDLR promote hepatic clearance of circulating LDL cholesterol, which reduces vascular lipid accumulation and subsequent plaque formation (118). Recent research demonstrates that extracellular vesicles derived from LDLR-overexpressing hepatocytes efficiently package and deliver functional LDLR transcripts to recipient cells. These mRNA molecules maintained remarkable stability after cellular uptake and were successfully converted into biologically active LDL receptors, establishing a new biological platform for therapeutic intervention in diabetic foot ulcers (120). Modulating miRNA cargo in extracellular vesicles demonstrates significant therapeutic potential for diabetes mellitus and its complications. Specifically, adipose-derived stem cell extracellular vesicles engineered to overexpress miR-132 exhibit remarkable wound-healing properties in diabetic conditions through three synergistic mechanisms suppression of local inflammatory responses, stimulation of neovascularization, and induction of M2 macrophage polarization (121). Engineered extracellular vesicles enriched with miR-31-5p show great therapeutic potential in diabetic wound repair by simultaneously promoting vascular network regeneration, epithelial tissue repair and controlled scar tissue formation. This multifaceted approach effectively addresses the impaired healing cascade characteristic of diabetic pathophysiology (122). Extracellular vesicles derived from mesenchymal stem cells (MSCs) hold considerable therapeutic promise for diabetic neuropathy, showing neuroprotective effects through a variety of mechanisms, including axonal regeneration, modulation of inflammatory responses, and improvement of neuronal function (115, 123). Genetic engineering approaches to enrich mesenchymal stem cell-derived extracellular vesicles with miR-146a significantly enhance their therapeutic potential. This modification potentiates the vesicles’ biological activity through improved anti-inflammatory and regenerative capacities, demonstrating superior efficacy compared to native extracellular vesicles (115, 123). Elevated expression of specific miRNA - including miR-133b, miR-126, and miR-26a - has been shown to enhance neural repair processes, offering novel therapeutic avenues for diabetic neuropathy. These regulatory molecules show potential for nerve repair through multiple mechanisms, including axonal regeneration, neuroprotection, and modulation of the local microenvironment (115, 123).

In summary, genetic engineering approaches for modifying extracellular vesicles and their parent cells to deliver specific molecular cargoes—particularly therapeutic miRNA—represent a transformative strategy for diabetes mellitus and its complications. These advances provide new mechanistic insights into vesicle biology and open the clinical application of engineered extracellular vesicles as a platform for targeted therapies, establishing a new paradigm for precision medicine in metabolic diseases.

#### 6.2.3 Delivery of therapeutic proteins via exosomes

Diabetic organ pathology frequently correlates with significant downregulation of heat shock proteins (Hsp), particularly in affected tissues. This defect in molecular chaperone activity impairs cellular stress responses and protein homeostasis, leading to diabetes-related tissue damage (124). To counteract this pathological defect, researchers engineered cardiomyocytes to overexpress Hsp20 to produce extracellular vesicles enriched with this protective heat shock protein. This targeted approach demonstrates the feasibility of modulating molecular chaperone content in extracellular vesicles for therapeutic applications in diabetic organ damage (124). Extracellular vesicles enriched with Hsp20 have demonstrated significant cardioprotective efficacy, ameliorating both functional and structural cardiac abnormalities in diabetic models. This evidence-based approach establishes a novel therapeutic paradigm for diabetes-induced cardiomyopathy through vesicle-mediated delivery of cytoprotective proteins (124).

Building on this approach, recent investigations have developed a novel composite therapeutic system by encapsulating protein hydrogels within extracellular vesicles. These engineered vesicle-hydrogel hybrids exhibit synergistic wound healing properties under diabetic conditions through a dual mechanism - the hydrogel component provides biomechanical support and cytoprotective scaffolding for tissue regeneration, while the vesicle delivery system ensures targeted transport and controlled release of therapeutic substances to the wound site. This biomimetic strategy capitalizes on the inherent biocompatibility of both components - the structural advantages of protein hydrogels and the natural delivery efficiency of extracellular vesicles - resulting in significantly accelerated wound closure rates in diabetic models (125).

In summary, the strategic engineering of extracellular vesicles and their mother cells through genetic modification and therapeutic loading (including Hsp20 and protein hydrogels) has created a transformative platform for diabetes and its complications. These advanced biotechnological approaches enable targeted molecular interventions, enhance tissue-specific repair mechanisms, and provide novel solutions for diabetes-associated organ pathology, representing a significant advancement in precision medicine for metabolic disorders.

### 6.3 Stem cell-derived exosome therapy for diabetic complications

#### 6.3.1 The role of exosomes in diabetic nephropathy

Mesenchymal stem cell-derived extracellular vesicles (MSCs-EXOs) demonstrate multi-faceted renoprotective effects in diabetic nephropathy through their diverse microRNA cargo. These vesicles preserve renal function via three synergistic mechanisms suppression of NOD2 pathway activation, attenuation of high glucose-induced podocyte apoptosis, and reduction of intrarenal inflammatory mediators. This tripartite action highlights their therapeutic potential for mitigating diabetes-induced kidney injury (126). miR-486-5p was delivered by MSCs-EXOs, which polarizes macrophages to an immunoregulatory M2 phenotype through precise molecular targeting. This microRNA-mediated regulation involves direct inhibition of PIK3R1 expression, subsequent downregulation of PI3K/Akt pathway activity, and subsequent attenuation of the renal inflammatory response. This mechanistic cascade highlights the sophisticated immunomodulatory potential of MSCs-EXOs in diabetic nephropathy (127). The microRNA miR-424-5p exerts potent renoprotective effects by specifically targeting the Yes1-associated transcriptional regulator (YAP1) in renal tubular epithelial cells. This molecular modulation achieves a dual therapeutic effect - inhibition of renal epithelial cell apoptosis and blockade of epithelial mesenchymal transition (EMT), which protects renal tissue structure and function (128). miR-125a exerts renoprotective effects under hyperglycemic conditions by directly targeting histone deacetylase 1 (HDAC1), leading to suppression of the HDAC1/endothelin-1 (ET-1) signaling axis. This microRNA-mediated regulation attenuates glucose-induced renal damage through epigenetic modulation of pathological signaling pathways (120). Meanwhile, miR-125b exhibited a dual regulatory function in renal cortical cells by silencing the expression of tumor necrosis factor receptor-associated factor 6 (TRAF6) and subsequently orchestrating a cytoprotective response characterized by enhanced autophagic flux and inhibition of apoptotic signaling. This microRNA-mediated modulation of TRAF6-dependent pathways represents a potential therapeutic target for preserving renal cellular homeostasis under pathological conditions (129).

Umbilical cord mesenchymal stem cell-derived extracellular vesicles (UC-MSCs-EXOs) demonstrate potent antifibrotic effects in diabetic nephropathy through targeted modulation of the Hedgehog signaling pathway. These exosomes specifically suppress smoothened (SMO) receptor expression, resulting in attenuation of renal tubular epithelial cell interstitial fibrosis and significant retardation of diabetes-induced renal fibrotic progression. This targeted pathway intervention highlights the therapeutic potential of UC-MSCs-EXOs for managing diabetic kidney pathology (130). Under hyperglycemic conditions, extracellular vesicle-derived miR-22-3p exerts dual renoprotective effects by suppressing NLRP3 inflammasome activation in podocytes and attenuating intrarenal inflammatory responses. This microRNA-mediated inhibition of the NLRP3 signaling pathway represents a potential therapeutic strategy for mitigating glucose-induced podocyte injury in diabetic nephropathy (131). miR-146a-5p orchestrates macrophage polarization toward the immunoregulatory M2 phenotype through targeted suppression of the TRAF6/STAT1 signaling axis. This microRNA-mediated immunomodulation attenuates pro-inflammatory signaling cascades and ameliorates renal inflammatory pathology, showing the significant therapeutic potential for inflammatory kidney disorders (132). Extracellular vesicles derived from human urine stem cells (USC-EXOs) exhibit glomeruloprotective effects through coordinated modulation of renal cellular homeostasis. These exosomes suppress mTOR pathway activation, regulate autophagic flux (as evidenced by increased LC3B-II conversion), and preserve glomerular filtration function in diabetic conditions. This multipronged mechanism highlights the therapeutic potential of USC-EXOs for mitigating diabetes-induced glomerulopathy (133). miR-16-5p from urine-derived stem cells (USCs) exerts renoprotective effects in diabetic nephropathy through targeted VEGF-A gene silencing. This microRNA-mediated regulation significantly attenuates podocyte apoptosis and ameliorates diabetes-induced renal injury by modulating the VEGF-A-dependent signaling pathway, highlighting its potential as a therapeutic target for diabetic kidney disease (134). Bone marrow mesenchymal stem cell-derived extracellular vesicles (BMSCs-EXOs) confer significant cytoprotection in diabetic nephropathy through their miR-30e-5p cargo. This microRNA specifically targets ELAV-like RNA binding protein 1 (ELAVL1), resulting in marked suppression of caspase-1-mediated pyroptosis and preservation of renal tubular epithelial cell viability under hyperglycemic conditions. This molecular mechanism highlights the therapeutic potential of BMSCs-EXOs for mitigating glucose-induced renal tubular injury (135). These extracellular vesicles demonstrate dual renoprotective mechanisms in diabetic nephropathy by downregulating key apoptotic executioner proteins (e.g., caspases-3/7), and suppressing pro-inflammatory mediators including TNF-α and NF-κB signaling pathways. This coordinated action significantly attenuates both apoptotic cell death and inflammatory-mediated renal injury, offering a multifaceted therapeutic approach for diabetic kidney disease (136). Multiple stem cell populations and their derived extracellular vesicles demonstrate significant therapeutic potential for diabetic nephropathy, largely mediated through their rich repertoire of regulatory miRNA. These vesicular miRNAs orchestrate pleiotropic protective effects by simultaneously modulating multiple pathological pathways involved in diabetic kidney injury.

#### 6.3.2 The role of exosomes in diabetic neuropathy

Mesenchymal stem cell-derived EXOs demonstrate dual therapeutic benefits in diabetic complications through their potent anti-inflammatory properties and pro-regenerative capacities. These extracellular exosomes facilitate vascular and neural network restoration while promoting functional neurological recovery. The underlying mechanism seems to involve the regulation of the mTOR pathway, in which exosome-mediated induction of autophagic processes simultaneously reduces the inflammatory response and enhances tissue repair mechanisms (123).

Extracellular exosomes derived from SIRT1-overexpressing bone marrow mesenchymal stem cells demonstrate potent neuroprotective effects by mitigating oxidative stress-induced neuronal damage and preserving mitochondrial integrity. This exosome-mediated protection substantially enhances functional neural recovery through SIRT1-dependent mechanisms that regulate cellular redox homeostasis and mitochondrial dynamics (89). These findings imply that selectively engineered mesenchymal stem cell-derived extracellular vesicles represent a promising therapeutic platform for both neuroprotection and neural functional restoration. The targeted molecular composition of these EXOs enables dual therapeutic capabilities: protecting neuronal populations from degenerative damage while actively facilitating neural repair processes.

The hyperglycemic microenvironment in diabetes impairs Schwann cell proliferation and compromises neurofilament protein homeostasis, contributing to progressive neural damage. Notably, adipose-derived stem cell extracellular vesicles exhibit therapeutic potential by stimulating Schwann cell regeneration and restoring neurofilament expression, offering a promising intervention for diabetic neuropathy (137). These findings establish a new therapeutic paradigm for diabetic neuropathy through an exosome-based intervention strategy that provides both mechanism-level insights and translational potential. The demonstrated ability to modulate Shewan’s cell dynamics and neurofilament protein regulation marks a significant advance in addressing the underlying pathophysiology of diabetic nerve injury.

In summary, EXOs secreted by mesenchymal cells show great potential in reducing inflammation, promoting the reconstruction of vascular and neural connections, and recovering neural function in diabetic individuals. At the same time, specific types of MSCs-derived EXOs have effects such as protecting nerve cells and promoting the proliferation of Schwann cells, offering new possibilities for the treatment of diabetes and its related complications.

#### 6.3.3 The role of exosomes in diabetic cardiovascular complications

MSCs-derived EXOs have significant protective effects on cardiac tissues in diabetic patients. These EXOs can alleviate inflammation, necrotic apoptosis, and tissue remodeling in the heart, effectively improving cardiac dysfunction caused by diabetes (138). This discovery offers a new perspective on the treatment of diabetic cardiovascular complications. Furthermore, EXOs derived from human umbilical cord MSCs also exhibit positive effects on heart function. They may reduce the expression of autophagy-related proteins in cardiomyocytes by activating the AMPK-Unc-51-like autophagy-activating kinase (ULK) 1 signaling pathway, thereby mitigating cardiac dysfunction induced by high-glucose conditions (139). This mechanism helps maintain the healthy state of heart cells and prevents further damage to heart function caused by diabetes. In addition to their direct impact on heart function, EXOs derived from human umbilical cord MSCs may regulate key physiological processes such as energy metabolism, oxidative stress, inflammation, and cell communication by maintaining the homeostasis of the Janus kinase (JAK)/STAT pathway, thus delaying diabetes-induced vascular remodeling (140). This finding reveals the multiple roles of EXOs in regulating diabetic-related cardiovascular physiological processes and provides strong support for the development of new therapeutic strategies.

In summary, MSCs-derived EXOs significantly improve diabetes-mediated cardiovascular dysfunction through mechanisms such as reducing inflammation, necrotic apoptosis, and tissue remodeling. Meanwhile, EXOs derived from human umbilical cord MSCs further protect heart and vascular health by activating specific signaling pathways and maintaining the homeostasis of key physiological processes. These discoveries offer new ideas and methods for the prevention and treatment of diabetic cardiovascular complications.

## 7 The translational application of engineered exosomes in the treatment of diabetic wounds

### 7.1 Pathophysiological mechanisms and clinical challenges in diabetic wound healing

Diabetic wounds, particularly diabetic foot ulcers (DFUs), represent a significant clinical challenge, with epidemiological studies demonstrating a lifetime incidence of 19-34% among diabetic patients and a 5-year amputation risk approaching 20% (141). The complex pathophysiology of impaired wound healing in diabetes involves three well-characterized yet interrelated pathological axes:

(1) Neuropathic Derangements:

The diabetic microenvironment induces significant modifications in the composition and function of neuronal-derived EXOs. These altered EXOs exhibit impaired intercellular communication capacity, disrupting critical neuro-regenerative pathways. Specifically, they interfere with Schwann cell migration and axonal regeneration, contributing to the characteristic loss of protective sensation in diabetic neuropathy (142). This neural dysfunction creates a permissive environment for wound development and impairs the neurogenic inflammatory response essential for initiating wound repair.

(2) Vascular Compromise:

Endothelial cell-derived EXOs in diabetes demonstrate substantial alterations in their molecular profiles. These modified EXOs contribute to microvascular dysfunction through several mechanisms: 1) dysregulation of angiogenic signaling pathways, 2) promotion of endothelial barrier dysfunction, and 3) impairment of vascular remodeling processes. The resultant chronic ischemia and impaired nutrient delivery significantly contribute to wound chronicity (143, 144).

(3) Persistent Inflammatory State:

The diabetic wound microenvironment is characterized by a sustained pro-inflammatory condition mediated through macrophage-derived EXOs. These EXOs maintain inflammatory signaling via complex cytokine networks, particularly involving TNF-α and IL-1β. The failure to transition from the inflammatory to proliferative phase represents a hallmark of diabetic wound pathophysiology (145).

### 7.2 Engineered exosomes strategies for diabetic wound management

Hypoxic preconditioning endows mesenchymal stem cell-derived nanovesicles (MSC-NVs) with enriched miR-210-3p cargo (146). Under hypoxic stress, cellular responses initiate complex adaptive mechanisms encompassing autophagic flux modulation, stress-response pathway activation, and energy homeostasis reprogramming (147).Crucially, hypoxia-inducible factors (HIFs) orchestrate transcriptional programs governing fundamental biological processes, including proliferation-apoptosis balance, metabolic adaptation, immune surveillance, and tumorigenic metastasis (139). Mesenchymal stromal cells (MSCs) demonstrate pronounced HIF-1α stabilization when subjected to oxygen deprivation. Studies confirm hypoxic preconditioning potentiates exosomal secretory capacity (148). Notably, chronic hypoxic microenvironments represent a pathological hallmark of diabetic ulcers. Consequently, EXOs engineered via this hypoxia-mimicking strategy exhibit inherent therapeutic potential for diabetic wound targeting.

### 7.3 Engineered exosomes strategies for wound healing

EXOs-based therapies have emerged as a transformative approach for wound repair, demonstrating efficacy across diverse preclinical models and early-stage clinical trials. Current strategies leverage engineered EXOs to address key pathological processes in chronic wounds, including impaired angiogenesis, excessive fibrosis, and persistent infection (149).

(1) Preclinical Advancements

In diabetic murine models, hypoxia-preconditioned hUC-MSCs EXOs delivered via hydrogel dressings enhanced vascular density by 300% and accelerated healing by 50% through miR-126-3p/VEGF axis activation (150). Similarly, ADSC EXOs overexpressing miR-29a normalized collagen III/I ratios (↑2.1-fold) and reduced scar formation by 60% in burn injuries by modulating TGF-β3/β1 balance (131). For infected wounds, BMSCs-EXOs loaded with silver nanoparticles (AgNPs) achieved 99% MRSA clearance while boosting epithelialization by 40% (151), highlighting the dual antimicrobial and regenerative potential of engineered EXOs.

(2) Clinical Progress

Phase II trials demonstrated that allogenic hUC-MSCs EXOs injections achieved 68% complete diabetic foot ulcer healing at 12 weeks—double the rate of standard care (32%) (152). In venous leg ulcers, autologous SVF-EXOs delivered via fibrin glue reduced wound area by ≥50% in 85% of patients, outperforming placebo (45%) (153). Early-phase burn trials confirmed EXOs safety, with lyophilized BMSCs-EXOs sprays shortening re-epithelialization time by 30% (127).

(3) Engineering Breakthroughs

Advanced modifications further enhance EXOs functionality: 1) Targeted delivery, iPSC-EC EXOs with CXCR4 overexpression and magnetic nanoparticles improved ischemic flap survival by 75% through 5-fold enhanced homing (154). 2) Gene editing, Treg EXOs displaying PD-L1 delayed skin graft rejection by 14 days via CD8+ T cell inhibition (↓80%) (155). 3) Combinatorial therapy, MSCs-EXOs co-loaded with vancomycin eradicated 99% of MRSA while tripling collagen deposition in porcine wounds (156) (**Table 2**).

**Table 2:** EXOs-based interventions in wound healing: preclinical and clinical evidence.

Category disease model	EXOs source	Engineering strategy	Delivery method	Key outcomes
Diabetic mouse full-thickness wound (150)	hUC-MSCs- EXOs	Hypoxic preconditioning (HIF-1α↑)	Hydrogel dressing	Angiogenesis↑300% Healing rate↑50% (miR-126-3p/VEGF)
Rat burn model (131)	ADSC EXOs	miR-29a overexpression	Intradermal injection	Col III/I ratio↑2.1-fold Scar area↓60%
Porcine infected wound (151)	BMSCs- EXOs + AgNPs	Sonication-assisted AgNP loading	Spray application	MRSA clearance↓99% 2, Epithelialization↑40%
Diabetic foot ulcer (Phase II) Allogenic (152)	hUC-MSCs- EXOs	Unmodified	Perilesional injection	12-week healing: 68% (EXOs) vs 32% (control)
Venous leg ulcer (Phase I/II) (153)	Autologous SVF-EXOs	3D culture-enhanced yield	Fibrin glue	Ulcer area↓≥50%: 85% (EXOs) vs 45% (placebo)
Mouse radiation-induced injury (154)	iPSC-EC EXOs	CXCR4↑ + magnetic nanoparticles	Magnetic targeting IV	Homing efficiency↑5-fold ROS clearance↑90%
Porcine wound + MRSA (156)	MSCs-EXOs + Vancomycin	Electroporation loading	Thermosensitive hydrogel	Bacterial clearance↑99%;Collagen deposition↑300% (synergy)

EXOs, exosomes; HIF-1α, hypoxia-inducible factor-1α; AgNPs, silver nanoparticles; ROS, reactive oxygen species.

## 8 Challenges and prospects of exosome therapy in diabetes mellitus

### 8.1 Experimental evidence for EV-based applications in diabetes and its complications

Based on a clinical trial, the level of miR-192 in urinary EXOs of patients with diabetic nephropathy is significantly elevated during normoalbuminuria and microalbuminuria stages (28). This suggest s that urinary exosome-derived miR-192 has great potential as a non-invasive biomarker for early diabetic nephropathy. It can reflect the stress response of renal cells and alterations in intercellular communication under diabetic pathological conditions. However, this study only demonstrated a correlation rather than causation. Further validation—such as knocking down or overexpressing miR-192— can be carried out to determine whether increased miR-192 levels cause renal injury or whether renal injury leads to a compensatory rise in miR-192. Moreover, the quantity of EXOs in urine is influenced by multiple factors such as water intake and renal function, posing a significant challenge in standardizing miR-192 levels to ensure reliable and reproducible results.

Regarding diabetic retinopathy, clinical studies have revealed that regenerative repair in the diseased retina is associated with intraocular MSCs releasing EXOs containing high levels of miR-222 (29). This discovery indicates a potential mechanism by which local stem cells may facilitate paracrine repair through EXOs, providing a theoretical basis for endogenous repair and offering important therapeutic implications. However, direct evidence that miR-222 in MSC-derived EXOs mediates this reparative effect remains lacking. Confirmation may require inhibiting miR-222 to observe whether the reparative effect is blocked. For therapeutic development, systemic administration may face challenges in crossing the blood-retinal barrier, while intraocular injection is invasive and carries risks. Therefore, efficient delivery of EXOs to intraocular lesion sites must be considered.

For diabetic patients, cardiomyocyte-derived EXOs are enriched with miR-302, which is believed to exert toxic effects on the circulatory system, leading to endothelial damage and myocardial remodeling (157). This trial implies that diseased cardiomyocytes remotely mediate endothelial injury and myocardial remodeling through the release of “toxic EXOs,” thereby enabling “self-amplification” of the disease. However, how universal is this finding among the diabetic population? Do all patients’ cardiomyocytes release EXOs rich in miR-302? Larger-scale clinical samples are needed to verify this.

EXOs derived from adipose-derived stem cells overexpressing Nrf2 have been shown to counteract senescence in endothelial progenitor cells. Additionally, they can suppress the expression of ROS and inflammatory factors, which helps maintain vascular function in diabetic foot ulcers and may delay ulcer deterioration (158). This elucidates the multifaceted functions of adipose-derived stem cell EXOs in delaying cellular senescence, counteracting oxidative stress, and reducing inflammation, providing strong theoretical support for the treatment of diabetic vascular complications. Nevertheless, these results were obtained from animal experiments, and animal responses may not fully represent effects in the human context.

EXOs from adipose tissue-derived MSCs can mitigate autoimmune destruction of pancreatic islet cells by increasing the number of regulatory T cells, thereby improving type 1 diabetes without influence on the lymphocyte proliferation index (159). This approach, which modulates T-cell balance rather than directly killing immune cells, may represent a safer and more physiological immune intervention strategy. This study was conducted on a mouse model, and there were significant differences between the murine and human immune systems. Thus, its efficacy and safety in humans remain unknown. This strategy may be effective in the early stages of the disease when a substantial number of β-cells remain, but its value may be limited to preventing recurrent autoimmune destruction after transplantation for patients in advanced stages who are entirely dependent on insulin.

It has been found that adipocyte-derived EXOs may induce insulin resistance by suppressing the expression of insulin receptor substrate-1 (IRS-1) and hormone-sensitive lipase (HSL) in adipocytes (12). This discovery explains how adipose tissue mediates insulin resistance via EXOs. However, it has limited direct therapeutic application, as simply clearing adipocyte-derived EXOs from patients is not feasible. Instead, it offers insights into blocking their production or signaling pathways (**Table 3**).

**Table 3:** Experimental evidence for EV-Based applications in diabetes and Its complications.

Condition	EV source/biomarker	Key clinical findings	Study type
Diabetic Nephropathy (DN) (28)	Urinary EXOs (miR-192)	Elevated urinary exosomal miR-192 levels were significantly increased in diabetic nephropathy (DN) patients, particularly among those with albuminuria, demonstrating potential as an early diagnostic biomarker for DN.	Clinical study
Diabetic Retinopathy (DR) (29)	miR-222	MSCs-exosomes enriched in miR-222 were associated with retinal regenerative changes, suggesting miRNA cargo mediates reparative effects.	Clinical study
Diabetic Foot Ulcers (DFU) (158)	Adipose-derived stem cells	Exosomes could possibly be used to alleviate the progression of DFUs in patients with diabetes by preventing the senescence of EPCs and inhibiting ROS and inflammatory cytokine expression to reduce inflammation, which would assist wound healing through improved vascularization	Animal study
Diabetic Cardiomyopathy (157)	Cardiomyocyte-derived exosomes (miR-320)	Diabetic cardiomyocytes release exosomes enriched with deleterious cargo (e.g., miR-320), which impair endothelial cell (EC) function, angiogenesis, and cardiac remodeling.	Clinical study
Type 1 Diabetes (T1DM) (159)	Adipose tissue-derived mesenchymal stem cells (AD-MSCs)	AD-MSC’s exosomes exert ameliorative effects on autoimmune T1DM through increasing regulatory T-cell population and their products without a change in the proliferation index of lymphocytes, which makes them more effective and practical candidates.	Animal study
Insulin Resistance (12)	Adipose-derived stem cells	the adipocyte-derived ELVs downregulated the expression of insulin receptor substrate-1 (IRS-1) and hormone-sensitive lipase (HSL) in adipocytes	Clinical study

**Table 3** synthesizes key experimental evidence supporting EV-based applications in diabetes. While promising biomarkers (e.g., urinary miR-192 for DN) and therapies (e.g., MSC-EVs for T1DM) have emerged, critical barriers still remain.

### 8.2 Standardization of preparation and quality control of exosomes

A paramount challenge in exosome-based therapy lies in the efficient and reproducible isolation and purification of EXOs from cell culture supernatants (160). Current exosome isolation strategies encompass ultracentrifugation (gold standard), immunoaffinity capture, size-exclusion chromatography, and polymeric precipitation (9). However, these methods are limited by suboptimal yield (<30% recovery), co-isolation of contaminants (e.g., protein aggregates), technical complexity, and inter-laboratory variability, ultimately compromising sample reproducibility (161). For clinical translation, establishing Good Manufacturing Practice (GMP)-compliant protocols is imperative to guarantee therapeutic-grade exosome production through standardized extraction, purification, and rigorous quality control (162). Emerging microfluidic-based and kit-based isolation methods show promise for scalable production while maintaining batch-to-batch consistency.

In addition, the surface markers, size distribution, and diversity of EXOs are also factors that affect the efficacy of EXOs. Thus, implementing multiparametric quality control — combining surface proteome profiling, small RNA sequencing, and functional potency assays — is essential to certify clinical-grade EXOs with defined biological activity and safety profiles (19).

### 8.3 Targeting and therapeutic effect of exosomes in diabetes

The intrinsic targeting capacity of EXOs constitutes a key advantage for their use as therapeutic delivery vectors (163). While EXOs naturally transport therapeutic cargos (e.g., RNAs, proteins) to recipient cells, their cell-type-specific targeting in diabetic conditions requires precision optimization (164). Engineering exosome surfaces via ligand conjugation (e.g., GLP-1 peptides), antibody decoration, or small-molecule attachment significantly improves their tropism for pancreatic β-cells and diseased endothelial cells. However, the molecular determinants underlying exosome targeting remain elusive, and strategies to minimize off-target uptake and reticuloendothelial clearance while maintaining specific cell recognition constitute a critical research gap (165).

The chronic efficacy and durability of exosome therapy require systematic evaluation (166). Despite demonstrated efficacy in ameliorating acute diabetic manifestations, robust clinical data on sustained effects, systemic toxicity, and host immunogenicity remain scarce (83). Thus, prospective studies must elucidate persistent therapeutic effects and synergistic potential of EXOs with standard antidiabetic agents (e.g., metformin, SGLT2 inhibitors) (167).

### 8.4 Drug regulation and ethical considerations of exosome therapy

As exosome-based diabetes therapies progress toward clinical translation, regulatory and ethical considerations have emerged as critical hurdles (162). As novel biotherapeutics, exosome manufacturing, clinical deployment, and oversight require internationally harmonized guidelines from competent authorities (FDA/EMA) (168). Currently, the absence of comprehensive quality control metrics for exosome production risks batch-to-batch variability in therapeutic efficacy and unanticipated toxicities. Thus, regulatory agencies must establish risk-based frameworks to certify exosome products’ safety, potency, and consistency.

Despite substantial therapeutic promise, *in vivo* pharmacokinetics, immunomodulatory effects, and off-target consequences of EXOs remain incompletely characterized (169). Ethical imperatives must be integrated into exosome therapy development, particularly regarding source material procurement and informed consent.

Furthermore, interindividual variability in treatment response necessitates personalized dosing strategies based on patient-specific factors (e.g., metabolic profile, comorbidity status) (170). Therefore, developing precision exosome therapies tailored to patients’ pathophysiological subtypes and complication spectra represents a pivotal research frontier (171).

## 9 Conclusion

Advances in exosome biology and biotechnology have positioned exosome-based therapies as promising approaches for diabetes and its complications. Priority research directions include: (1) GMP-compliant production optimization, (2) targeting precision enhancement, (3) host compatibility improvement, and (4) large-scale randomized clinical validation (34). Integrating precision medicine principles, engineered EXOs could emerge as key therapeutic platforms for patient-tailored diabetes management, offering enhanced efficacy and reduced adverse effects.

In summary, despite persisting challenges, ongoing technological innovations and standardization efforts will undoubtedly establish EXOs as transformative therapeutics for diabetes and its complications (172). This synthesis critically evaluates the dualistic role of EXOs in diabetes pathophysiology and their emerging diagnostic/therapeutic applications. While EXOs demonstrate significant mechanistic involvement in diabetic complications—mediating β-cell dysfunction via miR-320b (GDM) and miR-142-3p (T1DM), promoting renal fibrosis through TGF-β1 shuttling, and driving retinal neovascularization via VEGF-enriched cargo—their clinical utility faces substantial barriers.

## 10 Challenges and opportunities

### 10.1 GMP-compliant production optimization

Current exosome isolation techniques exhibit critical limitations in yield and reproducibility. Microfluidic platforms and AI-assisted quality control systems represent promising solutions to achieve GMP compliance. Standardized characterization protocols for vesicle composition and functionality remain an urgent need for regulatory approval.

### 10.2 Targeting precision enhancement

Although native exosomes demonstrate tissue tropism, precision targeting requires advanced bioengineering approaches. There are many challenges in this field, including (1) ligand-receptor specificity in diabetic microenvironments, (2) biodistribution control, and (3) dynamic targeting needs across complications. Emerging solutions incorporate biomimetic engineering, stimulus-responsive systems, and multimodal targeting strategies to enhance delivery efficiency.

### 10.3 Host compatibility improvement

The diabetic immune microenvironment presents unique obstacles, including accelerated opsonization and disease-stage variability. Innovative approaches focus on: (1) novel surface modifications to prolong circulation, (2) CRISPR-engineered cargo for multifunctional effects, and (3) patient-specific vesicle matching to minimize immune rejection.

### 10.4 Large-scale clinical validation

The field requires: (1) standardized Phase III trials with clinically relevant endpoints, (2) real-world evidence collection systems, and (3) combination therapy investigations. Establishing international consortia will be crucial to address regulatory gaps and accelerate clinical adoption.

## 11 Challenges and controversies

(1) Biomarker Specificity: Overlap in exosomal cargo (e.g., miR-21 in both diabetic nephropathy and retinopathy) complicates disease-specific diagnosis.
(2) Therapeutic Hurdles: Off-target effects of engineered EVs, immunogenicity of surface ligands, and scalability of GMP-compliant production.
(3) Conflicting Evidence: Discrepancies in EV functions (e.g., pro-inflammatory vs. regenerative roles) across studies due to heterogeneity in EV isolation methods and diabetic models.
(4) Research Gaps: Standardization of EV characterization, long-term safety data, and clinical validation of EV-based diagnostics.
